# Polycomb-mediated chromatin compaction weathers the STORM

**DOI:** 10.1186/s13059-016-0899-y

**Published:** 2016-02-25

**Authors:** Iain Williamson, Wendy A. Bickmore, Robert S. Illingworth

**Affiliations:** MRC Human Genetics Unit, IGMM, University of Edinburgh, Edinburgh, UK

**Keywords:** Fluorescence in situ hybridization, Polycomb, Chromatin compaction, Epigenetic, Super-resolution imaging, Stochastic optical reconstruction microscopy, Drosophila

## Abstract

A recent super-resolution imaging study by Boettiger et al. elegantly demonstrates that three epigenetically defined, and functionally disparate, chromatin states have distinct folding characteristics in *Drosophila* nuclei.

## Background

Chromatin provides a framework upon which the activity state of the underlying DNA sequence may be registered, modified or perpetuated. This is due, in part, to the addition of a range of chemical modifications to DNA and histones, which increase the information content of the genome to form the epigenome. However, the local packing state of chromatin is also thought to be related to the epigenetic state and an understanding of chromatin topology and three-dimensional (3D) chromatin architecture is therefore central to understanding gene regulation.

## Determining submegabase genome topology

Chromosome conformation capture (3C) techniques have shown that the genome is subdivided into topologically associating domains (TADs), which can range in size from tens to thousands of kilobases and which are defined by an extensive network of internal contacts. TADs appear to segregate genes and their distal regulatory elements into compartments that are crucial to gene regulation [1]. Within TADs, the chromatin state and the presence of DNA-binding proteins are indicative of the capacity of a genomic region and its constituent genes to be transcribed. Classically, inactive chromatin was considered to be more compact than active chromatin; indeed, this has been demonstrated biophysically for chromatin fibres purified from mammalian cells [2].

Investigation of chromatin compaction status in vivo, using DNA fluorescence in situ hybridization (FISH) has confirmed this [2]. Analysis of chromatin conformation by DNA FISH has mostly used conventional wide-field or confocal light microscopy or, more recently, super-resolution structured illumination microscopy (SIM), which generates super-resolution images from patterned light [3]. Photoactivation localization microscopy/stochastic optical reconstruction microscopy (PALM/STORM) super-resolution techniques use sequential activation and time-resolved localization of photoswitchable fluorophores to determine the precise position of single molecules from thousands of raw images, each with a different subset of emitting molecules. These methods can achieve a spatial resolution of <50 nm and have been mostly applied to determining the localization of protein molecules or membrane structures. However, Boettiger et al. [4] combined 3D STORM and DNA FISH with fluorescently labelled oligonucleotide probe pools to determine chromatin ultrastructure for different, epigenetically defined submegabase domains of the *Drosophila* genome.

## Epigenetic status defines distinct chromatin folding and spatial behaviour

**Fig. 1:**
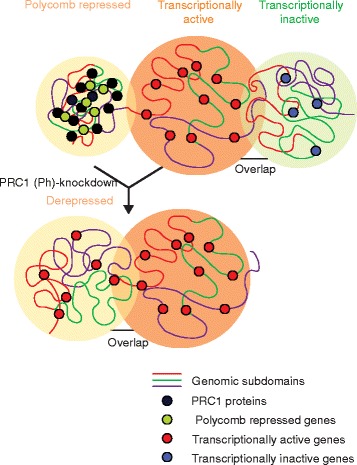
Epigenetically defined chromatin states have distinct conformations. Three chromatin domains identified in *Drosophila* nuclei are characterized epigenetically and functionally as polycomb-repressed, transcriptionally active and transcriptionally inactive (*top*). Repressed domains are the most compact and have minimal contact with other domains, properties that are lost upon disruption of polycomb repressive complex 1 (*PRC1*), which results in the activation of repressed genes (*bottom*). *Ph* polyhomeotic

Based on their histone modification state and bound complement of regulatory proteins, the regions studied were stratified as either transcriptionally active (histone H3 dimethylated lysine 4 (H3K4me2) or histone H3 trimethylated lysine 79 (H3K79me3)), polycomb-repressed (histone H3 trimethylated lysine 27 (H3K27me3) or polycomb group proteins) or inactive (depleted for modifications, polycomb components and transcriptional activators). Repressed chromatin marked by histone H3 lysine 9 methylation and the heterochromatin protein 1 family of proteins was not investigated. With probes ranging from 10 to 500 kb, the median volumes of transcriptionally active domains were consistently the largest, while polycomb-repressed domains were the most compact (Fig. 1). Consistent with observations from Hi-C analyses [1], the volume of the hybridization signals showed a power-law scaling behaviour with genomic length, but with different scaling exponents for the three domain types. The lowest chromatin packing density was detected for active regions, and the highest for the polycomb domains, consistent with previous FISH studies using conventional imaging in mammalian cells [2, 5, 6].

However, unlike the active and inactive domains, for which small subdivisions have similar scaling behaviours to their host domain as a whole, Boettiger et al. revealed a very different behaviour for polycomb-repressed chromatin. The hybridization signals from polycomb subdomains appear to traverse the entire volume of the whole host domain, indicating a high level of chromatin intermixing, which the authors confirmed by two-colour STORM. This contrasts with very limited intermixing of signals between active and polycomb-repressed domains when spatially juxtaposed along the chromosome. This observation is consistent with the spatial segregation of different TADs [7]; therefore, a particularly high level of chromatin intermixing within polycomb-repressed domains should be generally reflected in their pattern of intra-TAD signals as detected by 3C-derived techniques.

## Polycomb repressive complex 1 modulates chromatin architecture

Modelling of the imaging data suggested that, whereas the behaviour of the active and inactive chromatin domains conformed to that broadly expected from a fractal globule, the data from the polycomb-repressed domains did not. Instead, polycomb domain behaviour was better fitted to a sticky polymer model in which the chromatin is decorated with self-interacting monomers.

The compact chromatin state of polycomb-repressed domains in mammalian cells is caused by polycomb repressive complex 1 (PRC1), and not dependent on the catalytic activity of that complex [5]. In *Drosophila* cells, Boettiger et al. knocked down polyhomeotic (Ph), a PRC1 component whose sterile alpha motif (SAM) domain has known self-interacting properties. This resulted in the disruption of all architectural features of the repressed chromatin state, leading to chromatin decompaction, reduced intermixing within the domain, and de novo association with active chromatin domains. These findings are also in agreement with previous observations in *Drosophila*, showing that polycomb target loci are clustered within the nucleus into visible polycomb bodies. Distally located, polycomb-repressed loci separated by megabases of DNA coalesce within these bodies, albeit with varying frequencies depending on their chromosomal context [8]. However, a gap in our understanding of how chromatin architecture links mechanistically to the formation of these subnuclear structures still remains. STORM has recently been used to show that disruption of the Ph SAM domain leads to a marked dispersal of nanometre-scale polycomb foci, and a loss of distal contacts in chromosome conformation capture analyses [9].

A role for polycomb in scaffolding loci into defined bodies in mammalian cells is less evident. Recently, promoter capture Hi-C in mouse embryonic stem cells identified a marked enrichment for long-range intra- and inter-chromosomal interactions between polycomb target loci, centring primarily on the four paralogous polycomb-repressed *Hox* loci. Knockout experiments in combination with ChIP-seq (chromatin immunoprecipitation sequencing) identified that these interaction networks were both associated with and primarily dependent on PRC1 [10]. These findings suggest that PRC1 has a direct impact on metazoan nuclear organization by compacting target loci into tightly associated and spatially constrained functional chromatin domains.

## Moving forward

Taken together these results paint a picture of the distinctive 3D chromatin architecture characteristic of polycomb-repressed loci. However, the exact mechanism leading to the dissipation of these structures upon the loss of polycomb components, or forced differentiation, remains unclear. Loss of the aggregated PRC1 components could lead directly to the observed chromatin decompaction and altered structure (Fig. 1; *repressed* to *active*). Alternatively, mechanistically distinct phases of decompaction may exist, whereby the loss of polycomb interactions leads to a return to a basal or inactive chromatin conformation, followed by further decompaction to the active state as a result of coincident gene derepression (Fig. 1). The application of high-resolution imaging in conjunction with other emerging technologies will help to elucidate the cause–consequence relationship between polycomb-mediated repression and subnuclear chromatin organization.

## Abbreviations

3C: Chromosome conformation capture
3D: Three-dimensional
FISH: Fluorescence in situ hybridization
H3K4me2: Histone H3 dimethylated lysine 4
H3K27me3: Histone H3 trimethylated lysine 27
H3K79me3: Histone H3 trimethylated lysine 79
PALM: Photoactivation localization microscopy
Ph: Polyhomeotic
PRC1: Polycomb repressive complex 1
SAM: Sterile alpha motif
SIM: Structured illumination microscopy
STORM: Stochastic optical reconstruction microscopy
TAD: Topologically associating domain
